# The Impact of Migrations on the Health Services for Rare Diseases in Europe: The Example of Haemoglobin Disorders

**DOI:** 10.1155/2013/727905

**Published:** 2013-03-18

**Authors:** Michalis Angastiniotis, Joan-Lluis Vives Corrons, Elpidoforos S. Soteriades, Androulla Eleftheriou

**Affiliations:** ^1^Thalassaemia International Federation (TIF), 31 Ifigeneias Street, 2007 Strovolos, Nicosia, Cyprus; ^2^European Network for Rare and Congenital Anaemias (ENERCA), 08036 Barcelona, Spain; ^3^University of Barcelona, Hospital Clinic, Biological Diagnostic Centre, 08036 Barcelona, Spain; ^4^Cyprus Institute of Biomedical Sciences (CIBS), Department of Occupational and Environmental Medicine, 2042 Nicosia, Cyprus; ^5^Harvard School of Public Health, Department of Environmental Health, Environmental and Occupational Medicine and Epidemiology (EOME), Boston, MA 02115, USA

## Abstract

Migration from different parts of the world to several European countries leads to the introduction of haemoglobinopathy genes into the population, which creates several demanding needs for prevention and treatment services for Hb disorders. In this paper we examined the degree to which European health services have responded to such challenges and in particular to health services necessary to address the needs of patients with thalassaemia and sickle cell disease (SCD). Information on available services was obtained from international organizations, collaborated European project, and the Thalassaemia International Federation (TIF) Databases, which include information from published surveys, registries, field trips, and delegation visits to countries and regions by expert advisors, local associations, and other collaborators' reports. Results show that countries with traditional strong prevention and treatment programs are well prepared to face the above challenges, while others are urgently needed to address these problems in a systematic way. The Thalassaemia International Federation (TIF) is committed to monitor the progress, raise awareness, and support the promotion of more immigrant-oriented health policies to ensure their integration in society and their access to appropriate, adequate, and timely health services.

## 1. Introduction

Throughout history, poverty, land pressures, climate change, famine, war, and persecution have forced people to move from their homeland and in this context migration is not at all new. Migrants, like all people, carry with them personal health prints made up of ethnic and family disease susceptibilities and reflect the ways in which people and cultures have adapted to their physical environment and the mechanisms they have developed to deal with illness. As such, free population movements have always been considered important challenges to global health. Today as the gap between rich and poor countries is growing, people are moving faster and further, crossing vast climate and “disease zones”, being forced, in greater numbers, to seek work and better life elsewhere. At the same time richer countries are actively recruiting people to address their emerging labour needs while modern means of transportation and communication make it much easier for people to migrate while seeking better opportunities in life.

Population movements have had a major impact on disease epidemiology and public health. In the past, the main concern has been the spread of communicable diseases linked to poverty, suboptimal hygiene conditions, and lack of contemporary prevention programs and public health services. The more recent migration process observed in the end of the twentieth and dawn of the twenty-first century continues to contribute to the spread of communicable diseases but have in addition resulted in dramatic changes in the epidemiology of chronic diseases previously unknown or of low prevalence in host populations. These new “imports” represent a significant additional challenge to health services on a global scale.

Migrants experience a unique journey linked to the classical four phases of migration: premigration preparation, arrival, integration, and return. Without underestimating the significance of all stages of migration, during the arrival and integration phase, poverty and social exclusion are considered to exert their greatest effect on individual and group health outcomes. This is the period when the health of migrants is influenced by the availability, accessibility, acceptability, and quality of services in the new host environment. Health services may not be accessible because of linguistic, cultural, religious, and social barriers and this situation may sadly persist for many years after their establishment in the new host country.

The above were clearly evidenced in countries which traditionally hosted immigrants from countries where Hb disorders have been highly prevalent including UK, where in 2000 it was shown that despite available quality health services for prevention, screening, and care, there was only 50% uptake of such services by immigrants [1] and only 50% chance of survival of patients with *β*-thalassaemia major at the age of 35 [2]. The UK presents an example of a country where, today, third- or fourth-generation immigrants, from haemoglobinopathy prevalent countries, live and work and despite this, it was not until such data were analyzed that national strategies for these diseases were put in place.

The global geographical distribution of the haemoglobinopathy genes is today well documented [3, 4] and it is well known that in Europe, such genes are endogenous mainly in the populations of the south, especially in the countries of the Mediterranean basin and to a lesser extent in some of the countries of Eastern Europe. In contrast, these genetic traits are quite rare in the western, central, and northern countries of the European Continent. Migrants from the Middle East (ME), South East Asia (SEA), and other mainly malaria-endemic (or previously endemic) countries of the world have been moving, over the 20th century, to the western part of Europe. In addition migrants from the South, previously less affluent countries of Europe, have also been migrating towards West and North Europe. Such populations have now reached their second, third, or even fourth generation in these countries. In more current years, however, the immigration statistics are dramatically changing. There has been a substantial increase of population movements from ME, Asia, and Africa, much less from the south of Europe, and more from the Eastern European countries who have recently gained accession to the European Union (EU). Host countries in the last decade include mainly the fifteen old states of the EU [5]. These changes bring about considerable challenges to all systems of the host countries including most importantly their health services system.

In this study we have evaluated the degree to which European health services have responded to such challenges and in particular to health services necessary to address the control of rare anaemias and more specifically of haemoglobin disorders (Hb disorders): thalassaemia and sickle cell disease (SCD). We have focused on the compilation and analysis of the current status of services in specific European countries that appear to receive the majority of migrants from high-prevalence areas.

## 2. Methods

### 2.1. Countries

In this study we have included 12 European countries: Austria, Belgium, Cyprus, Denmark, France, Germany, Greece, Italy, The Netherlands, Spain, Sweden and the United Kingdom (UK).

### 2.2. Criteria

In order to examine the impact of Hb disorders on the health services, we examined the specific control programs for such health issues, which have literally been introduced through long-term and recent migration in almost every European country. Such assessment required the gathering of information on the following aspects of migration:

- the size and origin of each migrant group or ethnic minority in each host country and whether these are permanent or temporary workers/residents;
- the expected contribution of migrant groups to the haemoglobinopathy gene pool in the new host country;
- the marriage and reproductive behaviour of the new population groups versus the second, third (or more) generation of immigrants.

In addition, the services were evaluated having in mind the needs of those affected by such disorders. The services to be provided and planned for are those that aim at the best possible survival and quality of life of patients. These include preventive measures which will allow informed choices by risk couples from these communities. In considering the needs for health care services, the rarity of the conditions in many countries was taken into account. Service needs according to published best practice guidelines [6] were built mainly on knowledge and experience derived from successful control programs implemented in some countries. The characteristics of such programs include

- spread of community awareness, education, screening, and counselling,
- availability of possible solutions such as prenatal diagnosis or preimplantation diagnosis as available choices for informed couples,
- neonatal screening programs for sickle cell disease,
- system capacities to provide timely and accurate diagnosis,
- provision of medical and other care and monitoring,
- provision of multidisciplinary care in expert centres with effective networking of centres within and between countries to support primary and secondary level services,
- education of the medical and patients' communities.

In order to achieve the above objectives, appropriate public health and services planning were assessed regarding the existence of national policies to address the control of haemoglobin disorders, are regarded as essential. National policies may be disease specific as for example in a disease management programs (Cyprus, Italy, and Greece), under a general plan for rare diseases, or for chronic diseases in general (France).

### 2.3. Sources of Information

Since all Hb disorders and other rare anaemias are usually treated by the same clinical and public health services, it was possible for TIF, both through its own work and also through its partnership with national and international organizations and projects, to evaluate the current situation of services for Hb disorders and by extension for other rare congenital anaemias in European communities. In order to have a clear picture of the situation and to advocate for the development of services where they are most needed, TIF has developed, over the past few years, a global database for Hb disorders, which includes not only information on the burden of disease in each country, but also on available services and on the extent of access of patients to free and comprehensive care within national health systems. Having in mind the resources required for lifelong care, the existence of prevention measures as a tool to save and reallocate resources is also examined in this study. In order to estimate the magnitude of the problem the following parameters, numbers, and estimates were considered.

1. The number of immigrants in each European country, whose origin is from countries or populations with a thalassaemia and SCD carrier rate of more than 1%. The influx from countries with a low prevalence is not included in our estimates and the major source of the data is the Eurostat Population Database [7].
2. The carrier rate in each ethnic group which is derived from the carrier rate in the country of origin was used to estimate the number of carriers within each group. This was then added to the carrier rate of the indigenous population (for the low-prevalence countries the carrier rate is assumed to be 0.1% unless more accurate details are known) [5].
3. Carrier rates and other figures were taken from the Thalassaemia International Federation (TIF) Databases, which include information from published surveys, registries, field trips, and delegation visits to countries and regions by expert advisors, local associations, and other collaborators' reports. On occasions, reports were used from industry and national/regional health authorities and from relevant scientific conferences. In this database most African and American continents data are incomplete and were thus supplemented from the modell's almanac [8].
4. From the total carriers calculated as described above, the expected annual affected births were estimated according to the Hardy-Weinberg rule [9].
5. The disease burden was completed by recording the number of known patients in each country. The number was again obtained from TIF member associations, published information, and national registries wherever possible.

## 3. Results

Assessment of the quality of services currently available in each country has been made possible through the work of ENERCA and the funded project from the Executive Agency for Health and Consumers. This funding was provided for the preparation of the White Book on the identification of the criteria for haemoglobinopathy reference centres and networks in the European Union. This White Book includes several components such as the existence of national and regional health policies supporting services for haemoglobin disorders, the assessment of a comprehensive disease management policy for chronic and/or rare diseases. The existence of a national policy for prevention of thalassaemia including education, screening, counselling, and prenatal diagnosis, the establishment of a neonatal screening program for sickle cell disease, and the institution of a health insurance policy that allows free coverage for chronic disease patients, as compared to partial coverage that requires out-of-pocket expenses. 

In Table 1 we present several estimations for the number of Hb disorder carriers in different European countries. The results are presented to the nearest figures that were calculated on the available data on immigrant populations. It was assumed that Northern European populations have a thalassaemia carrier rate of 0.1% in their indigenous populations and no carriers of the sickle cell gene [3]. The importance is that in the countries where the prevalence is high in the indigenous population (Cyprus, Greece, and Italy) there are national policies to meet the needs of these disorders. In the rest of Europe, the proportion of immigrants is approximately similar, yet only the UK and France have disease-specific policies. The carrier frequency is rising most rapidly in Belgium and Spain where national planning is most urgently needed.

In Table 2 we present the disease burden from Hb disorders in each country under investigation in relation to the number of expected births and the number of known patients. In Table 3 we delineate the different health policies that constitute a prerequisite for the development and provision of diagnostic and clinical services to such patients, while in Table 4 we provide information regarding the most important prevention services available. In Table 5 we examine the different diagnostic and clinical services offered in each country such as designated treatment centres, reference laboratories, and the existence of specialized tests for treatment, including cardiac monitoring with magnetic resonance imaging (cardiac MRI T2*) for the assessment of cardiac iron load in regularly transfused patients and the transcranial doppler (TCD) to assess stroke risk in sickle cell patients.

**Figure 1:**
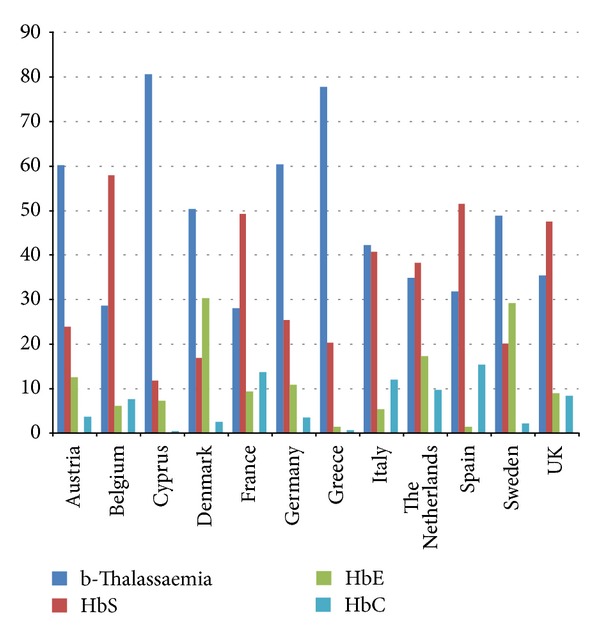
Relative proportion of carriers of Hb disorders among immigrant populations in selected European Countries. The denominator used included the total number of carriers of Hb disorders in each country.

**Figure 2:**
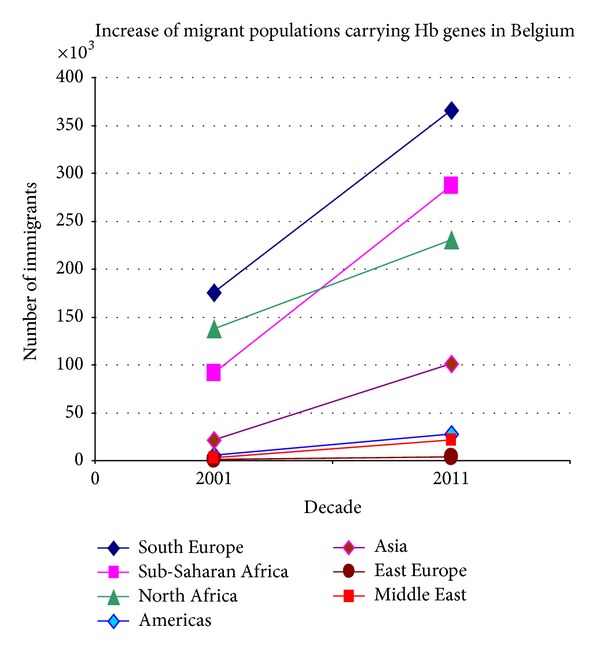
Increase in the number of immigrants carrying Hb disorders in Belgium between 2001 and 2011 according to the geographic region of origin.

**Figure 3:**
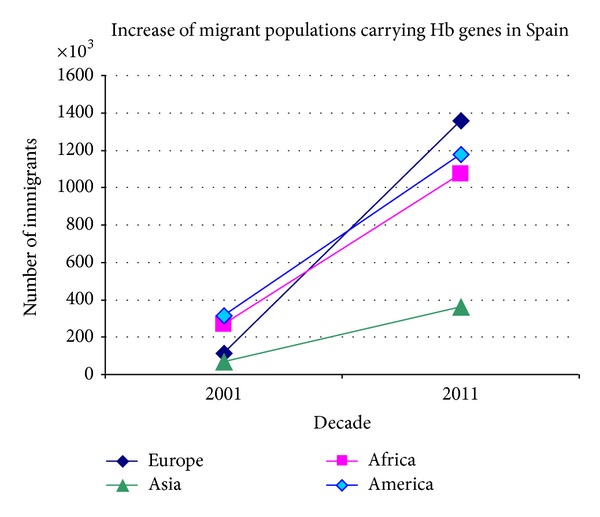
Increase in the number of immigrants carrying Hb disorders in Spain between 2001 and 2011 according to the geographic region of origin.

In Figure 1 we clearly see that thalassaemia and/or sickle cell disease genes are more common among the immigrant groups of European countries, and the gene predominance reflects the origin of the immigrant groups residing in each country. The increase in migrations from sub-Saharan Africa for example is seen in Belgium, France, Spain, Italy, and the UK, while other countries are influenced by migrations from Southern and Eastern Europe, West Pacific, and Asia such as Spain and France. The trend of accelerated influx of migrant populations from high-prevalence areas is presented in Figures 2 and 3, comparing the migrant populations between 2001 and 2011 in Belgium and Spain, respectively. Such trends are seen in most European countries and those of African descent seem to be increasing at a faster rate.

## 4. Discussion

The Thalassaemia International Federation (TIF) constitutes an international federation of 110 national patient support organizations from 60 countries around the world. Its mission is to support the development and implementation of national control and prevention programs and promote optimal management for Hb disorders. The ultimate goal is to secure equal access to quality health care for all patients with Hb disorders around the world.

TIF works in close and official relations with WHO headquarters and regional offices, promotes the two WHO's specific resolutions on thalassaemia and SCD, and supports WHO's plan of action for Noncommunicable Diseases (NCDs) and the inclusion of these diseases into the national policies for NCDs. TIF is also active in Europe, supporting the inclusion and promotion of Hb disorders into the community action and Council Recommendations on Rare Diseases [10–12]. Many other relevant World Health Assembly resolutions have been supported by TIF including those on the control of birth defects, viral hepatitis, health inequalities, social determinants of health and the haemoglobin disorders [13, 14].

In the above context, TIF supports the transposition of the EU Directive on Patients' rights for Cross Border Health Care into national legislation. Most importantly, it is involved in the promotion and networking of Reference Centres for Rare Diseases, including Hb disorders, which are amongst the prerequisites of the above directive. In addition, TIF operates on its own plan of activities, including its educational program, and participates in relevant EU projects such as Ithanet and ENERCA. Awareness and education of patients and health professionals is also achieved through the above information and organization networks.

With regards to immigrant groups information, there are several reasons why much or part of it may be approximations under- or overestimated. The quality of available data on carriers for example is often based on outdated, inappropriately sampled studies and/or small local surveys referring to selected groups rather than representing total populations. However we have used the best possible estimates derived not only from published data but also from TIF's database as described before.

The effect of migrations cannot be assessed only on the numbers of migrants. The behaviour of these groups in terms of marriage, reproduction, use of health services, permanency in the new host country, and other sociological factors also needs to be studied and considered. For example, intermarriage with the host population is certainly observed to occur as well as marriage with other ethnic minorities; however these parameters are not usually officially recorded in most countries. For instance, of the approximately 10.000 Filippino residents (potential carriers of both thalassaemia and HbE, which is nonexistent in the Cypriot population) in Cyprus in 2011, 7.702 are between the ages of 15 and 44 and 513 women were married in Cyprus in the last 4 years. These are females who come to Cyprus as domestic workers on a four-year contract. None was married to a countryman of theirs, 56% were married to a Cypriot, and 23% were married to a husband from another thalassaemia-prevalent country (the chances of a Cypriot-Filippino marriage producing an affected offspring are roughly 1 : 1.750). This is an indication that not all “temporary” migrants return to their home country. Such social variables cannot always be estimated and the example from Cyprus was chosen because of the readily available data from a small country.

Another variable whose effect can only be approximated is the contribution of nonregistered migrants who are estimated to be 1–4% of the population in Europe. This group will face all the problems of their “legal” counterparts and in addition will have to face even more difficulties in accessing the new host countries' health services because of fear of exposure. Where a chronic disease is concerned, the problems seem insurmountable and the help of NGOs which may step in to give assistance (http://www.nowhereland.info/) is usually very limited.

Another difficulty in assessing the importance of migration on the national and EU burden of these diseases is that the majority of European countries do not maintain comprehensive registries of patients, that include diagnosis, age distribution, location within a country, data on new cases, complication rates, and mortality data. One of the objectives of the current work is to highlight this problem and alert national health policy makers to the need for promoting national policies for Hb disorders and for the improvement of access and integration of immigrant patients to these services. Specific health services registries are considered essential to provide effective planning. In fact, it is quite difficult to understand how appropriate multidisciplinary services may be developed and provided in order to cover the needs of such patients in the absence of such information. Thus, one may argue that the lack or existence of a registry is indeed a major indicator of the quality of care in each country. Registries for Hb disorders exist currently only in France [15], Greece [16], Cyprus [17], Italy [18], and UK [19, 20]. Each of these referenced articles also demonstrates the usefulness of the derived information from national registries. The rest of the countries in this study provide estimates of the numbers of patients which, however, cannot be reliably confirmed.

In considering the services for chronic Hb disorders and their poor or limited uptake by the immigrant populations, one may elaborate on two major reasons. First there are constraints which originate from the social situation of the migrant groups in a host country, the low educational level especially of the new or first-generation migrants along with language and cultural barriers. Where hereditary disorders are concerned, there are also additional factors such as ignorance of the condition and its causes, including the possibility of carrier testing [21–24], social prejudice, and cultural and religious attitudes towards prevention and pregnancy termination. On many occasions, priority is rightly given to more acute problems related to family survival and occupational settlement in the new host country. The second factor is the response of health services in the host country to health problems which are initially and largely unknown and perhaps “foreign” to the indigenous population and not included in planning and policy making. The extent of the problem is usually only made known over time, since only a few such patients appear in hospital paediatric or adult departments and are considered as isolated cases.

Although, in more recent years, the importance of developing and implementing national programs in every EU member state by 2013 for rare diseases has been very much underscored, still to date surveys are generally lacking. Early diagnosis is often delayed and appropriate services for management are still largely absent or heterogeneously spread across, between and within European countries. In addition, strategic prevention of these disorders, in the vast majority of EU countries, is still absent at the national or regional level.

It is quite interesting that Mediterranean countries, which have a high prevalence of haemoglobinopathy genes in the indigenous population, are in recent years receiving immigrant groups from other high-prevalence countries. These countries, namely, Italy, Greece, and Cyprus, already have an infrastructure for the comprehensive management of these disorders with a long history of service development [25–27]. In these countries new affected births were drastically reduced by nationally controlled prevention programs mainly instituted by late 1980s. In recent years, however, the affected births are seen to be rising due to the poor response of the “new” immigrant groups as evidenced from the experience in Latio, Italy [28]. The question arises as to why these population groups do not take advantage of established prevention programs and health services. The most important reasons possibly relate to the lack of awareness of these immigrants for such programs due to the absence of previous experience from their countries of origin. In addition, the culture of carrier identification followed by possible prenatal diagnosis are not usually established in these population groups. Even when awareness is provided by the new host country's services, the decision of immigrants, at least in the first generation, is greatly influenced by their cultural and religious background linked to their country of origin [29, 30].

It is evident that, as a result of heavy past and continued migrations coupled with projections for future increase, the thalassaemias and sickle cell disease currently emerge as a visible public health problem in most of Europe. The new host countries of Europe appeared initially unprepared for this new yet increasing problem, and health authorities were unaware and unwilling to invest in services for what may have been viewed as a temporary situation. With time, the problem grew and treatment services started to be provided in few centres. Eventually prevention had to also be considered as the numbers of immigrants and potentially the number of annual affected births were increasing. The United Kingdom was the first among the Northern European states to develop a specific program and appropriate services [1], albeit initially not at the national level, almost concurrently with the endemic south. Other countries in the north have since followed, but many still have a long way to go in order to develop optimal and comprehensive services.

Europe had not experienced the need for developing genetic programs, which involve the total population to the same extent as the thalassaemia-endemic areas. The concept of community genetics has indeed introduced many concerns including ethical and legal challenges, complicated by the fact that these hereditary disorders are more common to immigrant populations with different cultures affecting the practical application of such programs. Demographic and epidemiological data are prerequisites and constitute a priority for development of national programs and services. The carrier rate may be the same as that of the country of origin, which is the method to calculate the figures presented in this report, but it would have been better estimated by local surveys, which could provide figures on annual affected births and the total and at-risk pregnancies. Such information could offer better or more accurate estimates for the planning of services as for example the size of the clinical services, the needed screening program, the number of prenatal diagnoses to be carried out, and the needs for couples' counseling [4]. In very few parts of Europe this process has been completed while in others it has yet to be faced.

There has been a tendency for immigrants to gather in specific areas, often forming “ghettoes”. For this reason, a second priority is micromapping through patient registries, with information on patient numbers and location so that needs for clinical and other services are rationally developed in geographically relevant locations. Clinical services should follow internationally accepted evidenced-based guidelines on which local, national, and regional standards of care could be integrated. It may be difficult for patients with rare anaemias in large countries to access expert centres of excellence as recommended by the comprehensive management programs of all currently available guidelines. This need can be met by networking and development of shared care with local centres as well as by physician training [31] within a comprehensive national policy for chronic and rare diseases. There is sufficient evidence, especially from survival data of various birth cohorts, that care in expert centres based on updated treatment protocols is associated with improved patient outcomes [17].

Designated treatment centres with access for all patients exist in Cyprus, Italy, Greece, and the UK (Table 4). In three other countries, only a portion of the patients have access to such centres. In addition, specialized laboratory and clinical tests necessary for addressing and preventing medical complications do not exist in the great majority of countries and even in reference or expert centres.

Acknowledging the limitation of the current paper, we would like to note that many of the research questions we posed in this study cannot be accurately answered because updates and/or reliable information may be unavailable. However, it was possible to build a picture of the current situation in Europe which may help raise awareness in the medical community and alert health authorities towards the particular needs in order to respond to the challenges that come with changes in the population demographics. Despite the rarity of the Hb diseases, their chronic nature and complexity result in a significant impact on health planning, which may lead to devastating and immense economic and other repercussions on individual patients and families. It must also be remembered that migrations are not constant but in fact are changing constantly in terms of size, direction, and impact, according to historical, environmental, political, and economic circumstances and therefore are difficult to follow. Because of this, the conclusions of this study reflect only on the very current situation and availability of health services for Hb disorders.

Financing services for Hb disorders is an important issue common to all chronic diseases' programs. Appropriate allocation of financial and human resources is essential for the sustainability of any such program. Some countries have adopted copayment systems, which may exert a difficult burden on migrants and low-income groups facing lifelong treatment and multidisciplinary care needs. In times of economic restraints, inequalities in access and health care services may be more likely to increase in an already global environment of severe inequalities in the health and social domains. Migrants with chronic diseases are particularly vulnerable to such situations.

The planning of programs for these genetic disorders is a public health exercise, which has not yet been adequately adopted in all European settings either as chronic disease policies or rare disease policies (Tables 3, 4, and 5). This is an unfortunate situation, since sufficient knowledge, experience, and expertise exist to adequately and effectively prevent and treat Hb disorders. In making plans, health authorities should also monitor the changes in the size and origins of incoming populations because changes may be significant over relatively short periods of time.

It is unquestionable that the prevention and treatment of thalassaemia and sickle cell disease patients have added to the health burden in many areas of the world by significantly contributing to budgetary constraints. However, governments around the world and particularly in Europe are already committed not only by having signed two important WHO specific resolutions [13, 14] but also by approving other EU directives on migrant health and recommendations on rare diseases. As a global NGO with responsibility and commitment for advocacy of patients' rights, TIF aims to continue its coordinated efforts to strengthen the support for the promotion of policies which will result in better outcomes for all patients with Hb disorders in the European and global arena. Each European country has a different approach to funding and providing cost-effective solutions to the multiple needs of chronically sick patients, especially among ethnic minorities. TIF is committed to monitor the progress, raise awareness, and support the promotion of more immigrant-oriented health policies to ensure their integration in society and their access to appropriate, adequate, and timely health services.

## Figures and Tables

**Table 1:** Estimations of the number of carriers in the countries studied.

Country	Total population	Total number of immigrants carriers of *β*-thalassaemia	Total number of carriers of b-thalassaemia in the indigenous population	Total number of immigrant carriers of HbE	Total number of immigrants carries of sickle cell	Total number of immigrant carriers of HbC	Carrier immigrants as a proportion of the total population	Carriers of Hb disorders as a proportion of the total population
(1) Austria	8210281	11842	8210	2453	4675	708	0.24%	0.34%
(2) Belgium	10438353	19403	10438	4073	39250	5169	0.65%	0.75%
(3) Cyprus	840407	3991	121019	354	583	20	0.58%	15%
(4) Denmark	5543453	6772	5543	4083	2277	330	0.24%	0.34%
(5) France	64057792	98219	64058	32607	172600	47884	0.54%	0.65%
(6) Germany	82329758	128419	82330	22955	53883	7135	0.25%	0.36%
(7) Greece	10737429	29289	837519	536	7626	183	0.35%	8.70%
(8) Italy	61261254	75748	2572972	9463	72870	21416	0.29%	6.50%
(9) The Netherlands	16715999	27656	16716	13751	30329	7703	0.47%	0.57%
(10) Spain	47042984	57257	715053	2434	92601	27796	0.38%	1.90%
(11) Sweden	9482855	21092	9483	12593	8720	912	0.46%	0.56%
(12) UK	63047162	107694	63047	27124	145038	25290	0.48%	0.58%

These results are the nearest figures that are calculated on the available data on immigrant populations. It was assumed that Northern European populations have a thalassaemia carrier rate of 0.1% in their indigenous populations and no carriers of the sickle cell gene. The importance is that in the countries where the prevalence is high in the indigenous population (Italy, Greece, and Cyprus), there are national policies to meet the needs of these disorders. In the rest of Europe the proportion of immigrants is approximately similar, yet only the UK and France have disease specific policies. The carrier frequency is rising most rapidly in Belgium and Spain where national planning is most urgently needed.

**Table 2:** Disease burden with the expected births and the number of known patients.

Countries	Expected thalassaemia births/1000 live births	Expected SCD births/1000 live births	Number of known or estimated patients with thalassaemia syndromes	Number of known or estimated patients with sickle cell syndromes
(1) Austria	0.0015	0.00008	NA	NA
(2) Belgium	0.002	0.0035	100	400
(3) Cyprus	5.5	0.0018	639	40
(4) Denmark	0.0018	0.00004	NA	NA
(5) France	0.0016	0.0018	571	10000
(6) Germany	0.0016	0.0001	1500	3000
(7) Greece	1.6	0.009	3241	1080
(8) Italy	0.46	0.11	7000	6000
(9) The Netherlands	0.0018	0.0008	250	750
(10) Spain	0.067	0.0011	113	>200
(11) Sweden	0.0025	0.00021	50	100
(12) UK	0.0018	0.0013	920	15000

**Table 3:** Health policies which support services.

Country	National register for Hb disorders	Hb disorders under national policy: chronic diseases	Hb disorders under national policy: rare diseases	Hb disorders under national policy: Blood diseases	National policy for prevention
(1) Austria	No				No
(2) Belgium	No	No	No	No	No
(3) Cyprus	Yes			Special policy	Yes
(4) Denmark	No	No	No	No	No
(5) France	Yes		Yes		Yes
(6) Germany	Initiated			Special policy	No
(7) Greece	Yes			Special policy	Yes
(8) Italy	Yes (regional)			Special policy	Yes
(9) The Netherlands	Initiated	No	No	No	Yes
(10) Spain	Regional	No	Yes	No	No
(11) Sweden	No	No	No	No	No
(12) UK	Yes			Special policy	Yes

**Table 4:** Policies and available prevention services.

Country	Carrier screening available	Carrier screening free	Neonatal screening	Prenatal diagnosis available
(1) Austria	No	No	No	No
(2) Belgium	Yes	No	Regional	Yes
(3) Cyprus	Yes	Yes	No	Yes
(4) Denmark				
(5) France	Targeted	Yes	Regional	Yes
(6) Germany	No	No	No	Yes
(7) Greece	Yes	Yes	No	Yes
(8) Italy	Yes	Yes	Regional	Yes
(9) The Netherlands	Yes		Yes	Yes
(10) Spain	Regional	Yes	Regional	No
(11) Sweden	No		No	No
(12) UK	Yes	Yes	Yes	Yes

**Table 5:** Diagnostic and screening services.

Country	Designated treatment centres serving all patients	Reference labs	Networks of labs	MRI T2*	TCD
(1) Austria	No	No	No	No	
(2) Belgium	Majority	Yes	No	No	Yes
(3) Cyprus	Yes	Yes	Yes	Yes	No
(4) Denmark	No	No	No		
(5) France	Yes	Yes	Yes	Yes	Yes
(6) Germany	Minority	Yes	Yes		Yes
(7) Greece	Yes	Yes	Yes	Yes	No
(8) Italy	Yes	Yes	Yes	Yes	Yes
(9) The Netherlands	No	Yes	Yes	No	
(10) Spain	Minority	Yes	Yes		
(11) Sweden	No	No	No		
(12) UK	Yes	Yes	Yes	Yes	Yes
